# COVID-19 News and Its Association With the Mental Health of Sexual and Gender Minority Adults: Cross-sectional Study

**DOI:** 10.2196/34710

**Published:** 2022-05-30

**Authors:** Kristen D Clark, Mitchell R Lunn, Athena D F Sherman, Hannah G Bosley, Micah E Lubensky, Juno Obedin-Maliver, Zubin Dastur, Annesa Flentje

**Affiliations:** 1 Department of Nursing University of New Hampshire Durham, NH United States; 2 Division of Nephrology Department of Medicine Stanford University School of Medicine Stanford, CA United States; 3 The PRIDE Study PRIDEnet Stanford University School of Medicine Stanford, CA United States; 4 Department of Epidemiology and Population Health Stanford University School of Medicine Stanford, CA United States; 5 Nell Hodgson Woodruff School of Nursing Emory University Atlanta, GA United States; 6 Department of Psychiatry University of California, San Francisco San Francisco, CA United States; 7 Department of Community Health Systems School of Nursing University of California, San Francisco San Francisco, CA United States; 8 Department of Obstetrics and Gynecology Stanford University School of Medicine Stanford, CA United States

**Keywords:** PTSD, posttraumatic stress disorder, anxiety, minority populations, vicarious trauma, tertiary trauma, COVID-19, pandemic, public health, sexual orientation, gender identity, mental health

## Abstract

**Background:**

Sexual and gender minority (SGM; people whose sexual orientation is not heterosexual or whose gender identity varies from what is traditionally associated with the sex assigned to them at birth) people experience high rates of trauma and substantial disparities in anxiety and posttraumatic stress disorder (PTSD). Exposure to traumatic stressors such as news related to COVID-19 may be associated with symptoms of anxiety and PTSD.

**Objective:**

This study aims to evaluate the relationship of COVID-19 news exposure with anxiety and PTSD symptoms in a sample of SGM adults in the United States.

**Methods:**

Data were collected between March 23 and August 2, 2020, from The PRIDE Study, a national longitudinal cohort study of SGM people. Regression analyses were used to analyze the relationship between self-reported news exposure and symptoms of anxiety using the Generalized Anxiety Disorder-7 and symptoms of COVID-19–related PTSD using the Impact of Events Scale-Revised.

**Results:**

Our sample included a total of 3079 SGM participants. Each unit increase in COVID-19–related news exposure was associated with greater anxiety symptoms (odds ratio 1.77, 95% CI 1.63-1.93; *P*<.001) and 1.93 greater odds of PTSD (95% CI 1.74-2.14; *P*<.001).

**Conclusions:**

Our study found that COVID-19 news exposure was positively associated with greater symptoms of anxiety and PTSD among SGM people. This supports previous literature in other populations where greater news exposure was associated with poorer mental health. Further research is needed to determine the direction of this relationship and to evaluate for differences among SGM subgroups with multiple marginalized identities.

## Introduction

Since the World Health Organization declared COVID-19 a pandemic in March 2020 [1], there have been more than 6 million deaths and 456 million confirmed cases worldwide (as of March 2022) [2]. In addition to the impacts of COVID-19 on physical health, mental health may be affected by direct (eg, infection with COVID-19, the threat of infection, or the loss of a loved one) and indirect (eg, witnessing the illness or death of others worldwide) stressors associated with the illness. As the numbers of sick and dead are counted and described across news outlets, individuals are exposed to an ongoing threat. This ongoing, repeated threat is meaningfully different from most traumatic stressors that occur once in a singular moment in time. Further, the ongoing stressors associated with COVID-19 pose a significant threat to vulnerable communities who have disparate exposure to trauma and associated mental illness [3,4]. For example, sexual and gender minority (SGM) people (ie, people whose sexual orientation is not heterosexual or whose gender identity varies from that which is traditionally associated with their sex assigned at birth, respectively) are particularly at risk of both trauma and poor mental health outcomes. Unique mental health disparities observed among this population [5] include high rates of depression [6,7], anxiety [8], and posttraumatic stress disorder (PTSD) [4,9]. These existing disparities in mental health outcomes may contribute to increased vulnerability to COVID-19–related stress among SGM people, resulting in worse mental health outcomes.

Compared to the general US population, SGM people may experience a higher stress burden related to COVID-19 due to pre-existing complex social and structural vulnerability. For example, SGM people are vulnerable to economic instability, such as higher rates of unemployment and poverty when compared to the general population [10,11]. This economic instability has been intensified among SGM people during the COVID-19 pandemic [12], as demonstrated by high rates of unemployment or reduced employment (eg, decreased hours) [13,14], increased likelihood of having trouble paying for housing expenses, and reporting overall financial hardship [14]. These conditions prompted the Centers for Disease Control and Prevention to caution about the risk for disproportionate effects from the COVID-19 pandemic among SGM people [15]. Initial research findings examining the mental health of SGM people during the COVID-19 pandemic show greater symptoms of anxiety and depression reported by those who had *no* symptoms before the emergence of COVID-19 [16]. However, the effects of COVID-19 on SGM people when compared to the general population have yet to be reported and remain an obstacle due to a lack of standardized data collection regarding sexual orientation and gender identity [17,18].

Increased exposure to stressors, like COVID-19, drives some individuals to engage with news and online information resources [19,20]. In a recent report, 74% of SGM individuals reported increased attentiveness to the news in response to COVID-19, compared to 68% of the general population; 60% of SGM participants indicated that they have “conducted their own research on the virus” compared to 45% of the general US population [14]. Increased engagement with COVID-19–related news may affect mental health outcomes by increasing exposure to traumatizing information or experiences. For example, those exposed to news during the COVID-19 pandemic experienced greater anxiety in relation to news exposure [21].

Public health experts have described the need to study COVID-19’s impact on the health of marginalized populations including SGM people [22,23]. Therefore, the purpose of this study was to investigate the relationship between COVID-19 news exposure (ie, time spent accessing COVID-19–related news resources) and symptoms of anxiety and PTSD among SGM individuals. We hypothesized that greater COVID-19 news exposure would be associated with greater symptoms of anxiety and, separately, that COVID-19 news exposure would be associated with greater symptoms of PTSD.

## Methods

### Participants and Procedures

Data were collected from the Coronavirus Impact Survey within The PRIDE Study, a longitudinal cohort study of SGM adults in the United States [24]. Eligible participants for The PRIDE Study meet the following inclusion criteria: identify as lesbian, gay, bisexual, transgender, queer, or another sexual or gender minority; are 18 years or older; reside in the United States or its territories; and are able to read and understand English. Participants are consented and enrolled through The PRIDE Study digital research platform. Participants in The PRIDE Study were recruited through multiple methods including through PRIDEnet Community Partners consisting of health, community, and other SGM-serving organizations within the United States; online through direct recruitment and advertising on social media and other venues; and in person at lesbian, gay, bisexual, transgender, queer community events. Further details about The PRIDE Study can be found elsewhere [24,25]. Data for the Coronavirus Impact Survey were collected from The PRIDE Study participants during the window of March 23 to August 2, 2020. Demographic items were merged from previous participant data within The PRIDE Study (eg, Annual Questionnaires).

### Ethics Approval

This study was approved by the institutional review boards of Stanford University (IRB-63400) and the University of California, San Francisco (IRB 18-26982).

### Measures

#### Demographics

Demographics measured included age, race/ethnicity, gender identity, sexual orientation, and the highest level of education completed. Participants could endorse all races or ethnicities that applied to them with a select-all-that-apply variable (ie, American Indian or Alaskan Native; Asian; Black, African American, or African; Hispanic, Latino, or Spanish; Middle Eastern or North African; Native Hawaiian or other Pacific Islander; White; and “none of these categories fully describes me”). Gender was measured by mutually exclusive categories in which participants self-selected the term that was most closely aligned with their gender identity (ie*,* cisgender woman, cisgender man, nonbinary, transgender man, transgender woman, or another gender identity). Sexual orientation was measured using mutually exclusive categories in which the participant self-selected the term that was most closely aligned with their sexual orientation (ie, asexual/demi/gray-ace, bi/pansexual, gay/lesbian, queer, straight/heterosexual, or another sexual orientation). Participants were included if they completed the Coronavirus Impact Survey items (ie, news exposure variable, the Generalized Anxiety Disorder 7 [GAD7], and the Impact of Events Scale-Revised [IES-R] scale; N=3079).

#### Symptoms of Anxiety

The GAD7 was developed to measure general anxiety disorder symptom severity [26]. Each of the seven items are measured on a four-point Likert-type scale where 0 indicates “not at all” and 3 indicates “nearly every day” regarding symptoms experienced during the past 2 weeks. The items are then summed and provide a range from 0 to 21 (*α*=.92). Diagnostic cutoff scores were applied to create an ordinal variable for our analyses (<5=no diagnosis of anxiety, 5-9=mild symptoms of anxiety, 10-14=moderate symptoms of anxiety, >14=severe symptoms of anxiety) [26].

#### Symptoms of Posttraumatic Stress

The IES-R was developed to measure PTSD symptom severity [27]. The IES-R is a self-report scale where participants indicated whether they experienced each item in the past 7 days within the context of COVID-19 experiences, defined as “hearing news about the virus, hearing about the experiences of others, having your own experience with symptoms, caregiving for someone with symptoms, or other experience related to the novel coronavirus.” Participants could respond to each of the 22 items using a Likert-type scale where 0 indicated “not at all” and 4 indicated “extremely” (*α*=.93). These items were summed and were dichotomized based on the diagnostic cutoff values where scores greater than 22 indicate presence of PTSD [27].

#### COVID-19 News Exposure

Participants were asked “How many hours a day do you watch or read the news for information about the novel coronavirus?” and provided a free-text box to report their estimation of time spent (in hours) engaging with COVID-19 news. Participants who answered fewer than 0 hours or greater than 24 hours were dropped from the final analysis (n=6). The variable was recoded as a four-level ordinal variable: less than 1 hour, 1 to less than 2 hours, 2 to 3 hours, and greater than 3 hours [21].

### Analysis

Descriptive statistics examined demographic variables within the total sample. The distributions of all variables were examined for outliers and missing data, which were dropped from the analyses (n=28). An ordinal logistic regression was used to evaluate the direct effects of news exposure with the GAD7 ordinal categories while covarying race/ethnicity, age, education, sexual orientation, and gender identity in the first model. In the second model, a logistic regression was used to evaluate the direct effects of news exposure with the IES-R dichotomous outcome (presence or absence of symptoms) while covarying race/ethnicity, age, education, sexual orientation, and gender identity. All analyses were run using STATA 15 (Statacorp) [28]. Standardized and unstandardized regression coefficients were compared with the alpha set at *P*<.05 (2-tailed).

## Results

### Sample Characteristics

Our sample included a total of 3079 SGM participants with a median age of 32.3 years (IQR 25.9-44.5 years; Table 1). Cisgender men comprised 27.8% (n=782) of our sample, 34.3% (n=965) were cisgender women, 18.5% (n=520) were nonbinary, 11.4% (n=322) were transgender men, 4.9% (n=965) were transgender women, and 3.2% (n=90) reported that their gender was not listed. Among the sample, 70.3% (n=2165) described their race or ethnicity as only White, 4.4% (*n*=135) described themselves as Hispanic/Latino/a, 3.6% (n=112) as Asian, 2.9% (n=89) as Black, and the remaining sample as additional races or ethnicities. A total of 24.4% (n=751) were multiracial. Our sample was highly educated: 42% (n=1292) indicated that they completed a graduate degree and 35% (n=1078) completed a 2- or 4-year college degree.

**Table 1:** Characteristics of The PRIDE Study’s Coronavirus Impact Survey (March 23 to August 2, 2020) sample (N=3079).

Variable		Value
Age (years), mean (SD)		36.7 (14.3)
**Race/ethnicity^a^, n (%)**		
	American Indian/Alaska Native	75 (2.44)
	Asian	112 (3.64)
	Black/African American/African	89 (2.89)
	Latino/a/Hispanic	135 (4.38)
	Middle Eastern/North African	27 (0.88)
	Multiracial	752 (24.42)
	Native Hawaiian/Asian Pacific Islander	6 (0.19)
	White	2408 (78.21)
	A racial/ethnic identity not listed	29 (0.94)
**Gender identity, n (%)**		
	Cisgender man	782 (27.8)
	Cisgender woman	965 (34.3)
	Nonbinary	520 (18.5)
	Transgender man	322 (11.4)
	Transgender woman	137 (4.9)
	A gender identity not listed	90 (3.2)
**Sexual orientation, n (%)**		
	Asexual/demisexual/gray-ace	262 (8.5)
	Bisexual/pansexual	865 (28.0)
	Gay/lesbian	1580 (51.4)
	Queer	344 (11.2)
	Straight/heterosexual	21 (0.7)
	Another sexual orientation	7 (0.2)
**Education level, n (%)**		
	Less than high school	18 (0.6)
	High school graduate, GED^b^, or some college	691 (22.4)
	College degree (2 or 4 years)	1078 (34.9)
	Graduate degree	1292 (42.0)

^a^Participants could select all options that applied.

^b^GED: General Educational Development.

### COVID-19 News Exposure and Anxiety Symptoms

Within our sample, 42% (n=1293) had mild anxiety symptoms, 34% (n=1047) had moderate anxiety symptoms, and 9% (n=277) had severe anxiety symptoms. The median GAD7 score was 8 (IQR 4-14). Each unit increase in COVID-19–related news exposure was associated with greater anxiety symptoms (odds ratio 1.77, 95% CI 1.63-1.93; *P*<.001; ie, GAD7; see Table 2 for full results).

**Figure 1:**
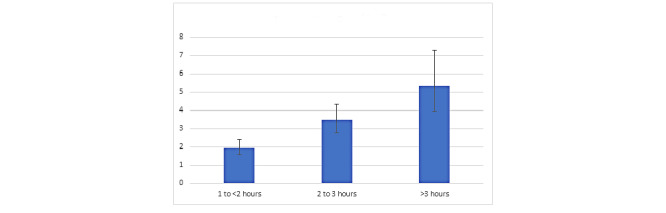
Odds of greater anxiety symptoms (GAD) with each level of COVID-19–related news exposure. GAD7: Generalized Anxiety Disorder-7.

Multimedia Appendix 1, Table S1 reflects the results of all variables of interest as well as indicator variables that were covaried. Figure 1 shows the relationship between COVID-19–related news exposure and anxiety symptoms for each of the different news categories.

**Table 2:** Results from ordinal logistic regression (GAD7) and logistic regression (IES-R) models that evaluated the relationship between COVID-19 news exposure and symptoms of anxiety (GAD7) and posttraumatic stress disorder (IES-R).

Model^a^	Odds ratio (SE)	95% CI	*z*	*P* value
COVID-19 news exposure and GAD7^b^	1.77 (0.08)	1.63-1.93	12.92	<.001
COVID-19 news exposure and IES-R^c^	1.93 (0.10)	1.74-2.14	12.48	<.001

^a^All models included the following covariates: age, education, sexual orientation, gender identity, and race/ethnicity.

^b^GAD7: Generalized Anxiety Disorder-7.

^c^IES-R: Impact of Events Scale-Revised.

### COVID-19 News Exposure and PTSD

**Figure 2:**
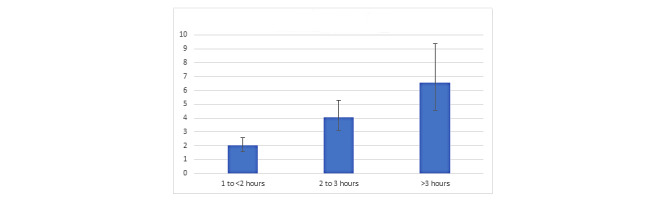
Odds of greater PTSD symptoms (IES-R) with each level of COVID-19–related news exposure. IES-R: Impact of Events Scale-Revised; PTSD: posttraumatic stress disorder.

A total of 57% (n=1755) of our sample met the threshold for presence of PTSD (score >22). The median of the IES-R score was 25 (IQR 15-37). Each unit increase in COVID-19–related news exposure was associated with 1.93 greater odds (95% CI 1.74-2.14; *P*<.001) of PTSD (ie, IES-R; see Table 2 for full results). Multimedia Appendix 1, Table S2 reflects the results of all variables of interest as well as indicator variables that were covaried. Figure 2 shows the relationship between COVID-19–related news exposure and PTSD for each of the different news conditions.

## Discussion

### Principal Findings

This study suggests that COVID-19–related news exposure is positively associated with greater anxiety and PTSD symptom severity among SGM people, consistent with previous findings within the general population [29-31]. Previous studies among the general population examined the impact of COVID-19–related media consumption on mental health, showing increased symptoms of anxiety and depression (particularly among those who were not symptomatic prior to the pandemic) [21,32,33]. This study extends this work to SGM populations, showing that COVID-19–related news exposure is substantially associated with greater symptoms of anxiety and PTSD.

It is important to note that these findings are correlational and, therefore, cannot establish directional causality. Nonetheless our study and others have found a correlation between COVID-19–related news exposure and mental health outcomes. For example, a recent study found that higher levels of anxiety at the start of the US pandemic were associated with greater time spent online [34], a frequent source of news, and time on smartphones accessing news sources [35]. A longitudinal study of participants with previously diagnosed PTSD reported greater news exposure, which was associated with greater symptoms of PTSD [36]. One explanation for our findings is that people with high anxiety or PTSD symptoms before the pandemic may be more likely to engage with COVID-19–related news, possibly to seek reassurance and cope with uncertainty. SGM individuals in our sample who engaged more with COVID-19–related news media (ie, as a reassurance-seeking behavior) may have higher baseline levels of anxiety or PTSD. Excessive reassurance-seeking behaviors prospectively predict symptoms of anxiety disorders, even when controlling for trait anxiety and intolerance of uncertainty [37]. Therefore, it is also possible that, even if news exposure was higher among those with greater trait anxiety, engagement with news may still have had an amplifying effect on anxiety symptoms. Longitudinal data from the pandemic where baseline anxiety is collected in addition to measures of news engagement could disentangle this relationship; however, a lack of systematic sexual orientation and gender identity measurement in large public data sets creates a significant barrier [38,39]. We do know that in a longitudinal study with a convenience sample of SGM people, worsening mental health symptoms such as depression, suicidality, and anxiety from April 2020, the start of US lockdown in some states, and at a 5-month follow up were observed [40]. Another study found that SGM people had worse depressive symptoms during the pandemic when compared to their pre–COVID-19 baseline depressive symptoms [16]. Within our sample, 85% of participants had at least mild anxiety symptoms at the time of data collection, whereas symptoms of anxiety in the general population between August 2020 to February 2021 were estimated to be 41.5% [41].

Social support is associated with improved mental health, while feelings of loneliness are associated with worse mental health symptoms [42]. COVID-19 has reduced in-person gatherings where people may seek engagement and support from others [43], leading to greater social media and online interaction. It also increases the potential for one to be exposed to COVID-19–related news [44]. Social media has been a source of misinformation throughout the COVID-19 pandemic [45,46], which may contribute to increased symptoms of anxiety. During the COVID-19 pandemic, SGM people reported lower social support compared to their cisgender heterosexual peers [13]. Virtual engagement may be sought as a substitute for in-person gatherings; however, social media use was positively correlated with symptoms of anxiety [47] and PTSD [48]. Therefore, changes in social support may offer one possible explanation for the deleterious impacts of COVID-19–related news exposure on mental health—particularly among already-marginalized communities such as SGM people.

### Implications of This Study

COVID-19 poses considerable risk to physical health, but the associated mental health risks are still emerging. Further evaluation of news exposure among populations with known mental health disparities is needed to address potential areas of increased psychological risk among individuals exhibiting symptoms, or worsening symptoms, of anxiety or PTSD. Investigation into the directionality of the relationship between COVID-19–related news exposure, anxiety, and PTSD symptoms is needed to identify possible mechanisms as well as opportunities for targeted interventions.

### Strengths, Limitations, and Future Directions

COVID-19 has presented us with unprecedented challenges globally, ranging from impacts to physical health to broad-reaching economic impacts. Our study expands on the emerging knowledge surrounding COVID-19 and its relationship to the mental health of SGM people. Our study illuminates how even indirect exposure to COVID-19 news was associated with mental health outcomes. Further, it points to the potential impacts on SGM people, a population for whom mental health disparities have been consistently observed. This offers opportunities for intervention where either news access can be altered or interventions to support mental health can be introduced. Further, we form these inferences based on a large sample of SGM people, who are frequently absent from analyses on traumatic events and associated mental health outcomes. However, there are several limitations to this study. This sample was obtained by convenience sampling, also limiting its representation of the broader SGM population and comparison to the general population. For example, our sample was highly educated with 42% (n=1292) of our sample reporting that they have a graduate degree. This varies from what is known about the education level of the broader SGM population, of whom 13% of SGM are estimated to have a graduate degree [49,50]. Self-report measurement may have resulted in social desirability bias with responses that are not representative of objective events. Our measure of news exposure did not differentiate between types of news; therefore, we were not able to determine how much of this time was spent on social media, direct from news agencies, or offline news sources (eg, traditional print media) that could impact the findings [30]. These sources of news may be related to our findings although participants may not include them in their time estimation based on whether or not participants perceive the source as news (eg, time spent on social media). Further, we cannot be sure whether the number of hours reported by participants was accurate; this is partially a function of the use of a free text entry for time as opposed to other methods for participant responses (eg, response choices provided in ranges). This results in some participants being dropped for responding with a number of hours greater than the possible 24 or less than 0 (n=6); however, this is a small number of the overall sample and unlikely to have impacted our findings. A certain level of bias is expected as to the meaning of exact, yet unlikely numbers (eg, 20 hours) are lost. Underlying health conditions that increase risk of COVID-19 complications could impact our findings as fear or concern of one’s health could increase anxiety, symptoms, or PTSD. Another important limitation to acknowledge is that while our sample includes diverse lived experiences (eg, sexual orientation, gender identity, race, and ethnicity), we do not address the way in which these social positions intersect and how that power differential may affect mental health outcomes and the way COVID-19–related news may be accessed and internalized. As recent reports have shown, the effects of COVID-19 have been disproportionate among people of color and certain SGM groups [13,51]. Future work in this area should involve mixed methods research so that qualitative data can contextualize these experiences [52].

Future work is needed in several areas. Analysis that differentiates the types of news consumption and whether there are differences in their relationship with anxiety and PTSD would help to identify more specific recommendations for protective mental health behaviors. Further, considering the important role of social support and how social media is both a source of social support but also a common news source necessitates further work. Along these lines, future studies should examine the relationships between problematic smartphone use and mental health symptoms—particularly within marginalized populations. A recent report indicated that the association between reassurance-seeking behaviors and problematic smartphone use may be a key mechanism in the maintenance of anxiety and depression [37,53]. Additionally, examination of baseline anxiety and PTSD symptoms would help determine if some groups are more at risk of worsening mental health outcomes than others.

### Conclusions

This study expands the available evidence supporting an association between news exposure and symptoms of anxiety and PTSD during the COVID-19 pandemic by identifying this relationship among a sample of SGM people. Opportunities exist for clinicians working with individuals to identify possible coping strategies, limit news and social media consumption, refer to safe means of social support, or incorporate psychoeducation about media consumption to reduce the accompanying stress caused by news exposure. As causality cannot be inferred from our findings, evaluation of systems of mental health access for existing anxiety and PTSD symptoms are necessary to ensure continuity of care. Further research exploring the effectiveness of online or social media community-based support and its role as a potential moderator between news stressor exposure and mental health symptoms is needed.

## Appendix

Multimedia Appendix 1 Supplementary tables.

## Abbreviations

GAD7: Generalized Anxiety Disorder 7
IES-R: Impact of Events Scale-Revised
PTSD: posttraumatic stress disorder
SGM: sexual and gender minority
