# Supplementary material for: COVID-19 News and Its Association With the Mental Health of Sexual and Gender Minority Adults: Cross-sectional Study
Source: JMIR Public Health Surveill. 2022 May 30;8(5):e34710. doi: 10.2196/34710 (PMC9153913; doi:10.2196/34710)
Supplement: Multimedia Appendix 1 [file publichealth_v8i5e34710_app1.docx]

**Multimedia Appendix 1.** Supplementary tables.

Table S1. Results from ordinal logistic regression models that evaluated the relationship between COVID-19 news exposure and symptoms of anxiety (GAD-7) with covariates.

| Model Variables | Odds Ratio | CI^a^ | *P* value | |
| --- | --- | --- | --- | --- |
| COVID-19 news exposure | 1.77 | 1.63, 1.93 | <.001 | |
| Age | 0.97 | 0.97, 0.98 | <.001 | |
| Education |  |  |  | |
| High school diploma or trade school | 0.69 | 0.27, 1.75 | .433 | |
| College degree (2- or 4- year) | 0.62 | 0.25, 1.58 | .319 | |
| Graduate degree | 0.48 | 0.19, 1.22 | .123 | |
| Sexual Orientation |  |  |  | |
| Asexual | 1.06 | 0.52, 2.18 | .866 | |
| Bisexual | 1.58 | 0.80, 3.15 | .191 | |
| Gay | 1.24 | 0.62, 2.45 | .545 | |
| Queer | 1.48 | 0.74, 2.99 | .272 | |
| Straight (ref) | - | - | - | |
| Gender Identity |  |  |  | |
| Cisgender man (ref) | - | - | - | |
| Cisgender woman | **2.61**^b^ | **2.15, 3.17** | **<.001** | |
| Non-binary | **3.23** | **2.54, 4.11** | **<.001** | |
| Transgender man | **2.50** | **1.91, 3.27** | **<.001** | |
| Transgender woman | **2.42** | **1.72, 3.40** | **<.001** | |
| Another gender identity | **2.65** | **1.75, 4.00** | **<.001** | |
| Race or Ethnicity |  |  |  | |
| American Indian or Alaskan Native | 1.12 | 0.73, 1.71 | .599 | |
| Asian | 0.78 | 0.54, 1.13 | .183 | |
| Black, African American, African | 1.11 | 0.74, 1.66 | .613 | |
| Hispanic, Latino, Spanish | 0.75 | 0.54, 1.04 | .080 | |
| Middle Eastern, North African | 1.36 | 0.68, 2.74 | .387 | |
| Native Hawaiian, Pacific Islander | 0.22 | 0.04, 1.25 | .088 | |
| White | **1.32** | **1.09, 1.60** | **<.01** | |
| Another race or ethnicity | **2.46** | **1.23, 4.95** | **<.05** | |
| ^a^Confidence Interval (CI)  ^b^Bolded indicates significance *P*<.05 | | | |  |

Table S2. Results from logistic regression models that evaluated the relationship between COVID-19 news exposure and symptoms of PTSD (IES-R) with covariates.

| Model | Odds Ratios | CI^a^ | *P* value |  |
| --- | --- | --- | --- | --- |
| COVID-19 news exposure | **1.93**^b^ | **1.74, 2.14** | **<.001** |  |
| Age | **0.98** | **0.98, 0.99** | **<.001** |  |
| Education |  |  |  |  |
| High school diploma or trade school | 1.04 | 0.34, 3.18 | .944 |  |
| College degree (2- or 4- year) | 0.95 | 0.34, 3.18 | .930 |  |
| Graduate degree | 0.75 | 0.25, 2.30 | .617 |  |
| Sexual Orientation |  |  |  |  |
| Asexual | 0.33 | 0.56, 1.93 | .217 |  |
| Bisexual | 0.45 | 0.08, 2.59 | .369 |  |
| Gay | 0.38 | 0.07, 2.22 | .284 |  |
| Queer | 0.53 | 0.09, 3.10 | .481 |  |
| Straight (ref) | - | - | - |  |
| Gender Identity |  |  |  |  |
| Cisgender man (ref) | - | - | - |  |
| Cisgender woman | **2.61** | **2.15, 3.17** | **<.001** |  |
| Non-binary | **3.23** | **2.54, 4.11** | **<.001** |  |
| Transgender man | **2.50** | **1.91, 3.27** | **<.001** |  |
| Transgender woman | **2.42** | **1.72, 3.40** | **<.001** |  |
| Another gender identity | **2.65** | **1.75, 4.00** | **<.001** |  |
| Race or Ethnicity |  |  |  |  |
| American Indian or Alaskan Native | 1.12 | 0.73, 1.71 | .599 |  |
| Asian | 0.78 | 0.54, 1.13 | .183 |  |
| Black, African American, African | 1.11 | 0.74, 1.66 | .613 |  |
| Hispanic, Latino, Spanish | 0.75 | 0.54, 1.04 | .080 |  |
| Middle Eastern, North African | 1.36 | 0.68, 2.71 | .387 |  |
| Native Hawaiian, Pacific Islander | 0.22 | 0.04, 1.25 | .088 |  |
| White | **1.32** | **1.09, 1.60** | **<.01** |  |
| Another race or ethnicity | **2.46** | **1.23, 4.95** | **<.05** |  |
| ^a^Confidence Interval (CI)  ^b^Bolded indicates significance *P*<.05 | | | | |
